# p62 is linked to mitophagy in oleic acid-induced adipogenesis in human adipose-derived stromal cells

**DOI:** 10.1186/s12944-018-0733-5

**Published:** 2018-06-04

**Authors:** Ruixia Zeng, Yan Fang, Yibo Zhang, Shuling Bai

**Affiliations:** 1 Department of Tissue Engineering, School of Fundamental Sciences, China Medical University, Shenyang, 110001 China; 2 Department of Anatomy, College of Basic Medical Sciences, Jinzhou Medical University, Jinzhou, 121001 China; 3 Liaoning Key Laboratory of Follicular Development and Reproductive Health, Jinzhou, 121001 China; 4 Department of Pathogenic Biology, College of Basic Medical Sciences, Jinzhou Medical University, Jinzhou, 121001 China

## Abstract

**Background:**

Obesity is closely related to the abnormal differentiation of adipocytes, which are subjected to high plasma levels of free fatty acids (FFAs). As the most abundant FFA in the bloodstream, oleic acid (OA) has the ability to induce adipogenic differentiation in human adipose-derived stromal cells (hADSCs). Recently, p62, an autophagy mediator, has been shown to play a role in obesity and adipose tissue metabolism. Therefore, the aim of this study was to investigate the roles of autophagy and mitochondrial function at different stages of OA (in combination with insulin and dexamethasone)-induced adipogenesis in hADSCs.

**Methods:**

The hADSCs were incubated with OA, insulin, and dexamethasone after pretreatment with autophagy inhibitors or knockdown of p62 with shRNA. The adiposeness level was then analyzed by oil red O staining in the cells. The related proteins or mRNA levels were detected by western blot analysis or quantitative real-time polymerase chain reaction (PCR).

**Results:**

Treatment with 80 μM OA (substituted for isobutylmethylxantine; IBMX) for 10 days successfully induced hADSCs to adipocytes. During OA-induced adipogenesis, autophagy was induced, with an increased LC3II/I ratio on day 3 and a decreased protein level of p62 on and after day 3. Inhibition of autophagy with 3-methyladenine (3MA) at the early stage (day 0 to day 3) of differentiation, but not at the middle or late stage, significantly decreased OA-induced adipogenesis; while knockdown of p62 with shRNA significantly promoted adipogenesis in hADSCs. Moreover, the copy number of mtDNA (the ND1 gene) and the protein level of TOM20, a mitochondrial membrane protein, were increased following OA treatment, which was related to the stability of mitochondria. Interestingly, knockdown of p62 increased the mito-LC3II/I and cyto-LC3II/I ratios by 110.1% and 73.3%, respectively. The increase in the ratio of mito-LC3II/I was higher than that of cyto-LC3II/I. Furthermore, p62 knockdown-enhanced adipogenesis in hADSCs was abolished by inhibiting mitophagy with cyclosporine A.

**Conclusions:**

These results suggested that p62 plays a protective role in adipogenesis of hADSCs through regulating mitophagy.

## Background

Obesity is characterized by excess adipose tissue growth probably due to the increased proliferation and differentiation of adipose-derived stromal cells (ADSCs), in addition to adipocyte hypertrophy [1, 2]. Besides, obesity, which is correlated with insulin resistance, is associated with increased plasma levels of free fatty acids (FFAs) [3–5]. Oleic acid (OA) is the most abundant FFA in the bloodstream. Studies have shown that 3 T3-L1 murine preadipocytes are able to be induced to accumulate lipid droplets in a serum-free medium supplemented with OA without the use of induction medium, including isobutylmethylxantine (IBMX), insulin, and dexamethasone [6, 7]. In addition, OA promotes the formation of triglyceride-rich lipid droplets and induces autophagy in hepatocytes [8]. Unlike other induction medium components, IBMX is a chemical that is not present in the human body. Therefore, in this study, OA was used as a substitute for IBMX to induce adipogenic differentiation in human ADSCs (hADSCs), which may more closely mimic human physiological conditions.

Autophagy is upregulated in adipose tissue from obese individuals and is correlated with the degree of obesity, visceral fat distribution, and adipocyte hypertrophy [9]. Recent studies have demonstrated that inhibition of autophagy by RNA interference against autophagy related 5 (Atg5) or Atg7 blocks adipogenic differentiation in 3 T3-L1 preadipocytes and in adipose tissue [10]. Consistently, pharmacological inhibition of autophagy prevents body weight gain and fat mass expansion, protecting against metabolic syndrome components such as glucose intolerance and insulin resistance. p62, a multifunctional protein and an important mediator in the autophagy–lysosome pathway, may play a role in obesity and adipose tissue metabolism [11–13]. In addition, p62 gene-knockout mice develop obesity and insulin resistance as well as show fat accumulation in the white adipose tissue, slow basic lipid hydrolysis, and increased lipid synthesis. However, whether p62 regulates adipocyte differentiation and the underlying mechanism are not clear. The p62-deficient mice also show metabolic changes, suggesting that p62 promotes a negative energy balance by inhibiting adipogenesis and favoring energy combustion [11]. This study will address the role of p62 and its relationship with autophagy in adipogenic differentiation of hADSCs.

Mitophagy is the term for mitochondrial selective autophagy. A specific receptor in the autophagic vacuole membrane can be identified by damaged mitochondria and then targeted degradation happens; this process is very important to maintain a normal number and function of mitochondria. The adipose differentiation process is accompanied by the formation of lipid droplets and the synthesis of triglycerides. Mitochondria are key to fatty acid oxidation, so this study will provide further understanding of the relationship between mitophagy and p62 after identifying the basic function of autophagy in hADSC adipose differentiation. It will illustrate the molecular mechanism of human adipocyte differentiation and provide a new way to identify novel drug targets for obesity.

Based on the above discussion, we built an OA-induced hADSC model and knocked down p62 in this model with lentiviral shRNA to explore the effects of p62 and mitophagy on the adipogenesis of hADSCs.

## Methods

### Culture and differentiation of hADSCs

All experiments were reviewed and approved by the Institutional Review Board of China Medical University. Thigh adipose tissue was obtained from females (30–35 years old) during liposuction surgeries. Isolation of hADSCs and differentiation into adipocytes were conducted as described previously [14]. Briefly, tissue was minced and enzymatically digested for 45 min in a buffer containing 0.1% collagenase I. The digested tissue was then filtered through 100-μm mesh and pelleted by centrifugation at 600×*g* for 5 min. hADSCs were grown in plastic dishes with low-glucose Dulbecco’s modified Eagle medium supplemented with 10% fetal bovine serum (FBS) in an atmosphere of 5% CO_2_ and 100% humidity at 37 °C. For each experiment, three parallel cultivations were prepared. Two days after reaching confluence (day 0), differentiation was initiated by switching to differentiation medium containing 10% FBS, 10 μg/mL insulin, 1 μM dexamethasone, and 50 μΜ, 80 μM, or 100 μM OA (O1008, Sigma-Aldrich, St. Louis, MO, USA).

### Oil red O staining and quantification of intracellular lipids

After adipogenic induction, cells were washed twice with phosphate-buffered saline (PBS) and fixed in 4% paraformaldehyde at room temperature for 30 min. Next, the cells were stained with an oil red O solution (60% oil red O stock solution and 40% H_2_O) for 15 min and then washed three times with PBS. The cell phenotype was observed under a light microscope (Olympus Provis BX41). For quantification, intracellular lipids were extracted using isopropanol, and the absorbance was measured at a wavelength of 500 nm [15].

### p62 lentiviral silencing

hADSCs were infected with lentivirus encoding p62 shRNA (GCAGATGAGGAAGATCGCCTT) or scrambled control shRNA, according to the manufacturer’s instructions. Lentiviral particles were purchased from GeneChem (Shanghai, China).

### Immunofluorescence

Cells were fixed in 4% paraformaldehyde, permeabilized in 0.25% Triton X-100, and blocked with 5% normal donkey serum. Next, the cells were incubated with rabbit anti-microtubule-associated protein light chain 3 (LC3) B monoclonal antibody (L7543, Sigma-Aldrich) in a cold room overnight and stained with goat anti-rabbit IgG Alexa Fluor 488 antibody (SAB4600389, Sigma-Aldrich) at room temperature for 1 h. The cells were subsequently viewed under a fluorescence microscope (TE2000-U, Nikon, Japan).

### Western blot

To prepare total cell lysates, monolayers of hADSCs were scraped with RIPA buffer containing protease inhibitors and phosphatase inhibitors. To separate mitochondrial and cytoplasmic fractions, the cells were scraped in mitochondrial isolation buffer. The collected cells were then mechanically disrupted. Cell slurries were centrifuged at 600×*g* for 5 min. The supernatant fractions were collected and centrifuged again at 8000×*g* for 15 min to pellet the mitochondria. The supernatant fractions represented the cytosolic fraction. The mitochondrial pellet was washed by resuspension in mitochondrial isolation buffer, spun again at 8000×*g* for 15 min, and lysed in RIPA buffer. Equal amounts of protein were separated in 4–20% Tris-glycine sodium dodecyl sulfate–polyacrylamide gel electrophoresis gels (Dalian TaKaRa) and transferred to nitrocellulose membranes. Membranes were blocked in 5% nonfat dry milk in Tris-buffered saline for 1 h at room temperature and then incubated with primary antibody diluted in 5% nonfat dry milk overnight at 4 °C [16]. The primary antibodies used were as follows: β-actin (Abp50151, Abcam, Cambridge, MA, USA), LC3, peroxisome proliferator activated receptor γ (PPARγ; 101,700–500, Abcam), p62 (P0067, Sigma-Aldrich), dynamin-related protein 1 (DRP-1; ABP51203, Abcam), mitofusin 1 (MFN-1; SAB2106161, Sigma-Aldrich), and mitochondrial outer membrane 20 (TOM20; HPA011562, Sigma-Aldrich). Membranes were washed in Tris-buffered saline containing Tween 20 (TBS-T) and incubated with horseradish peroxidase-conjugated anti-rabbit IgG secondary antibody (1:2000, Sigma) at room temperature for 1 h. The membranes were washed again in TBS-T, developed with an ECL Substrate (K820–50, Abcam), and imaged using a GelDoc XR System. Densitometry of the probed bands was analyzed by Image J2x.

### Quantitative real-time polymerase chain reaction (PCR)

Total RNA was extracted from cells using TRIZOL® Reagent (Promega), according to the manufacturer’s instructions. cDNA was synthesized using a First-Strand cDNA Synthesis Kit (Dalian TaKaRa). Real-time PCR was carried out with an ABI PRISM 7300 real-time PCR machine. The fold-change in gene expression relative to the control was calculated by the 2^-ΔΔCT^ method. The experiments were repeated three times, and every sample was assayed in triplicate. The primers used in the experiment are listed in Table 1.

**Table 1:** Sequences of primers used for real-time PCR

Gene	Primer (5′ → 3′)	
	Forward	Reverse
PPARG	AGCCTCATGAAGAGCCTTCCA	ACCCTTGCATCCTTCACAAGC
ACTB	ACTCTTCCAGCCTTCCTTCC	GTACTTGCGCTCAGGAGGAG
ND1	CCCTAAAACCCGCCACATC	GAGCGATGGTGAGAGCTAAGGT
GAPDH	ATGGGGAAGGTGAAGGTCG	GGGGTCATTGATGGCAACAATA

### Determination of mitochondrial DNA (mtDNA) copy number

The mtDNA copy number per cell was quantified as the amplicon ratio from the PCR with specific primers against a gene (NADH dehydrogenase subunit 1; Nd1) encoded by mtDNA and a gene (GAPDH) encoded by nuclear DNA. Total DNA was isolated from the cells using a DNA Extraction Kit (Omega). Quantification of mitochondrial and genomic genes was performed by the quantitative PCR method using the primer sequences listed in Table 1. The 2^-ΔΔCT^ method was performed as described in the previous paragraph. The ratio of 2^-ΔΔCT^ mito/2^-ΔΔCT^ nuclear was calculated for the combination of determined mitochondrial and nuclear genes.

### Statistical analysis

All data were expressed as the mean ± standard deviation (SD). Comparisons of data among groups were performed by one-way analysis of variance, and differences between two groups were analyzed by the Student–Newman–Keuls test using SPSS 17.0 software. A *p*-value of less than 0.05 was considered to be a significant difference.

## Results

### OA-induced adipogenesis of hADSCs

**Fig. 1:**
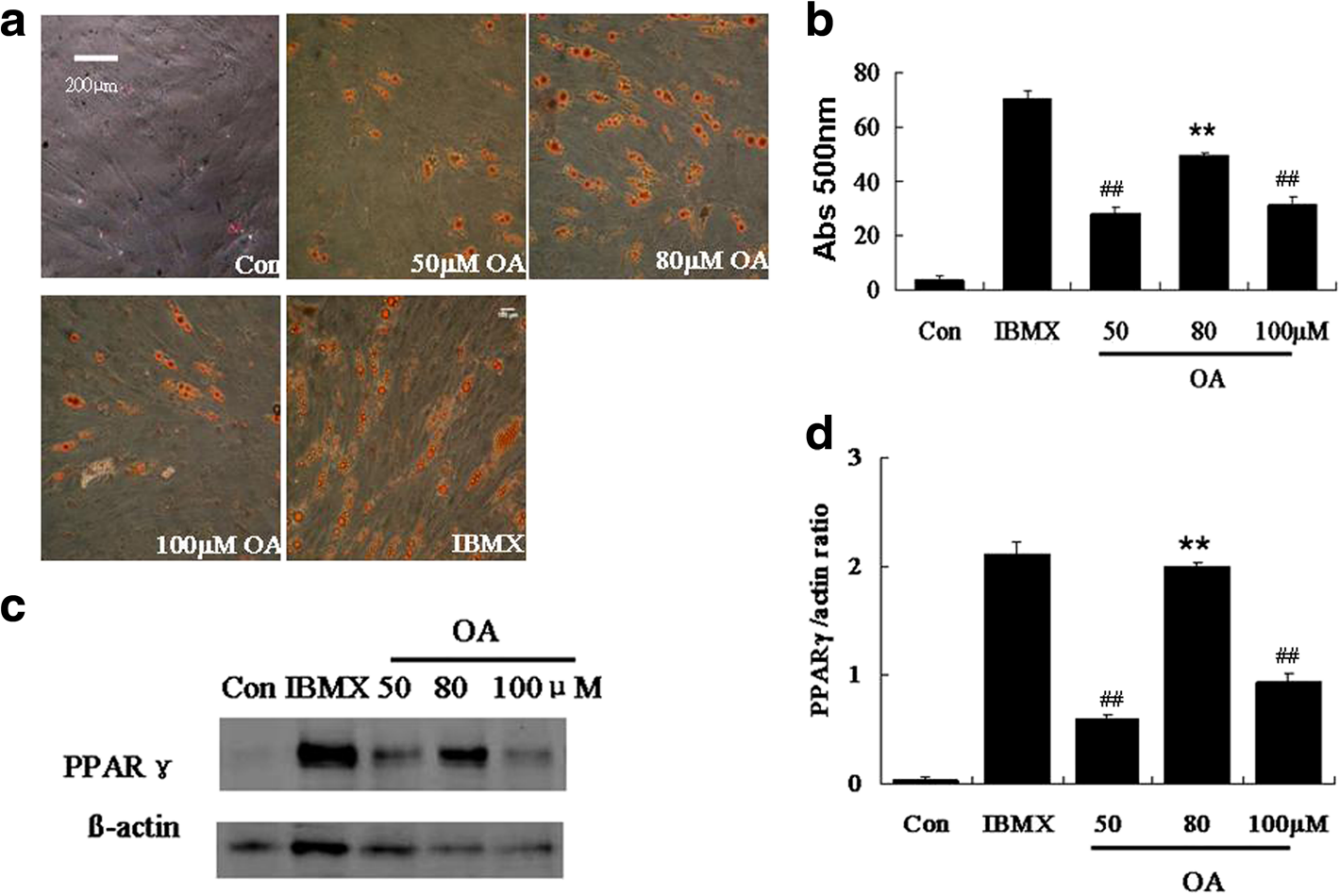
Adipogenesis of hADSCs induced by oleic acid (OA) in combination with insulin and dexamethasone. Cells were exposed to various concentrations of OA (50, 80, and 100 μM) or IBMX in combination with insulin and dexamethasone for 10 days. **a** Intracellular lipids were visualized by oil red O staining at day 10. **b** Quantification of intracellular lipids at the end of the incubation period (day 10). Briefly, oil red O-stained lipids were extracted with isopropanol, and the absorbance at 500 nm was determined by spectrophotometry. **c** and **d** The PPARγ protein expression level was analyzed by western blot at day 10. Data are shown as the mean ± SD (*n* = 3). **p < 0.01 vs. control, ^##^*p* < 0.01 vs. IBMX

Adipogenesis was measured after treatment with different concentrations (50, 80, and 100 μM) of OA in combination with insulin and dexamethasone. We found that OA, which was substituted for IBMX, successfully induced hADSCs to adipocytes, with visible cytoplasmic lipid droplets that were stained red by oil red O (Fig. 1a). In the group treated with 80 μM OA, the intracellular lipid content (Fig. 1b) and the protein expression level of PPARγ (Fig. 1c–d) were the highest among the three groups treated with different concentrations of OA (*p* < 0.01). Based on these results, 80 μM OA was chosen for the subsequent experiments.

### Autophagy was induced during adipogenesis of hADSCs

**Fig. 2:**
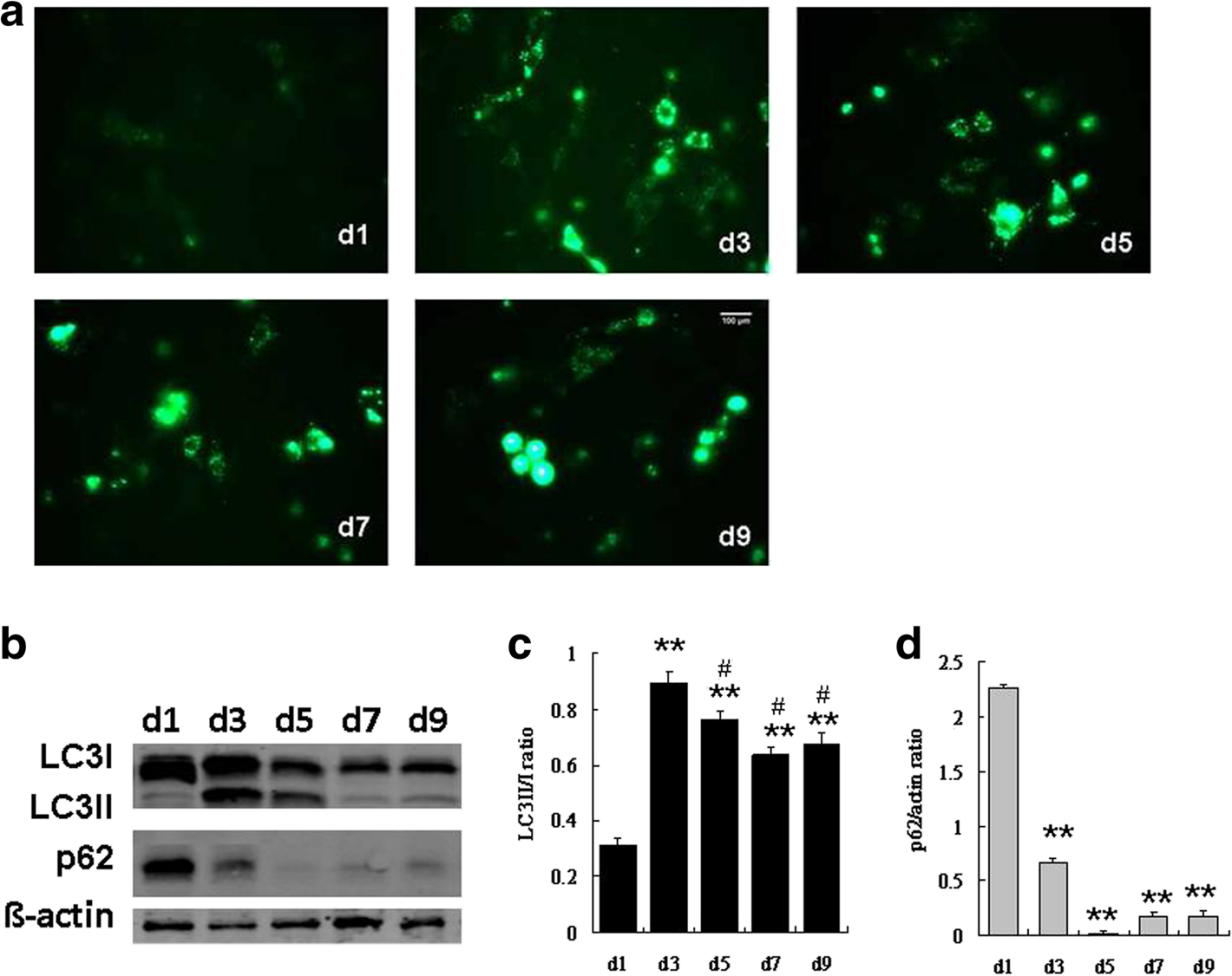
Autophagy at different stages of oleic acid (OA)-induced adipogenesis in hADSCs. **a** Representative immunofluorescence staining of LC3 in hADSCs at days 1, 3, 5, 7, and 9 of adipogenesis. **b** Western blot of LC3 and p62 in hADSCs during differentiation. **c** Quantification of the LC3II/I ratio. **d** Quantification of the p62 protein level. Data are shown as the mean ± SD (*n* = 3). ^**^p < 0.01 vs. d1, ^#^*p* < 0.05 vs. d3

To examine whether autophagy is involved in OA-induced adipogeneses of hADSCs, we detected the expression of the autophagy marker molecule LC3 at different time points [17, 18]. Immunofluorescence staining of LC3 showed that punctiform accumulation of LC3 II was increased from day 1 to day 3, while it decreased from day 4 to day 7, and then it returned to the undifferentiated level at day 9 (Fig. 2a). Western blot analysis showed that the LC3II/I ratios were higher during adipogenesis, compared with that on the first day (Fig. 2b–c), indicating that high autophagic levels were maintained throughout the whole differentiation process. The LC3II/I ratio on day 3 was significantly higher than that on the following days, and there was no significant difference in the LC3II/I ratios among days 5, 7, and 9 (Fig. 2b–c). p62, a key factor of autophagy, was also detected during adipogenesis. The protein level of p62 was the highest on day 1 and then decreased during the later phase of differentiation (Fig. 2b and d), suggesting that it might play a vital role in the adipogenesis of hADSCs.

### Inhibition of autophagy at the early stage of differentiation in hADSCs prevented adipogenesis

**Fig. 3:**
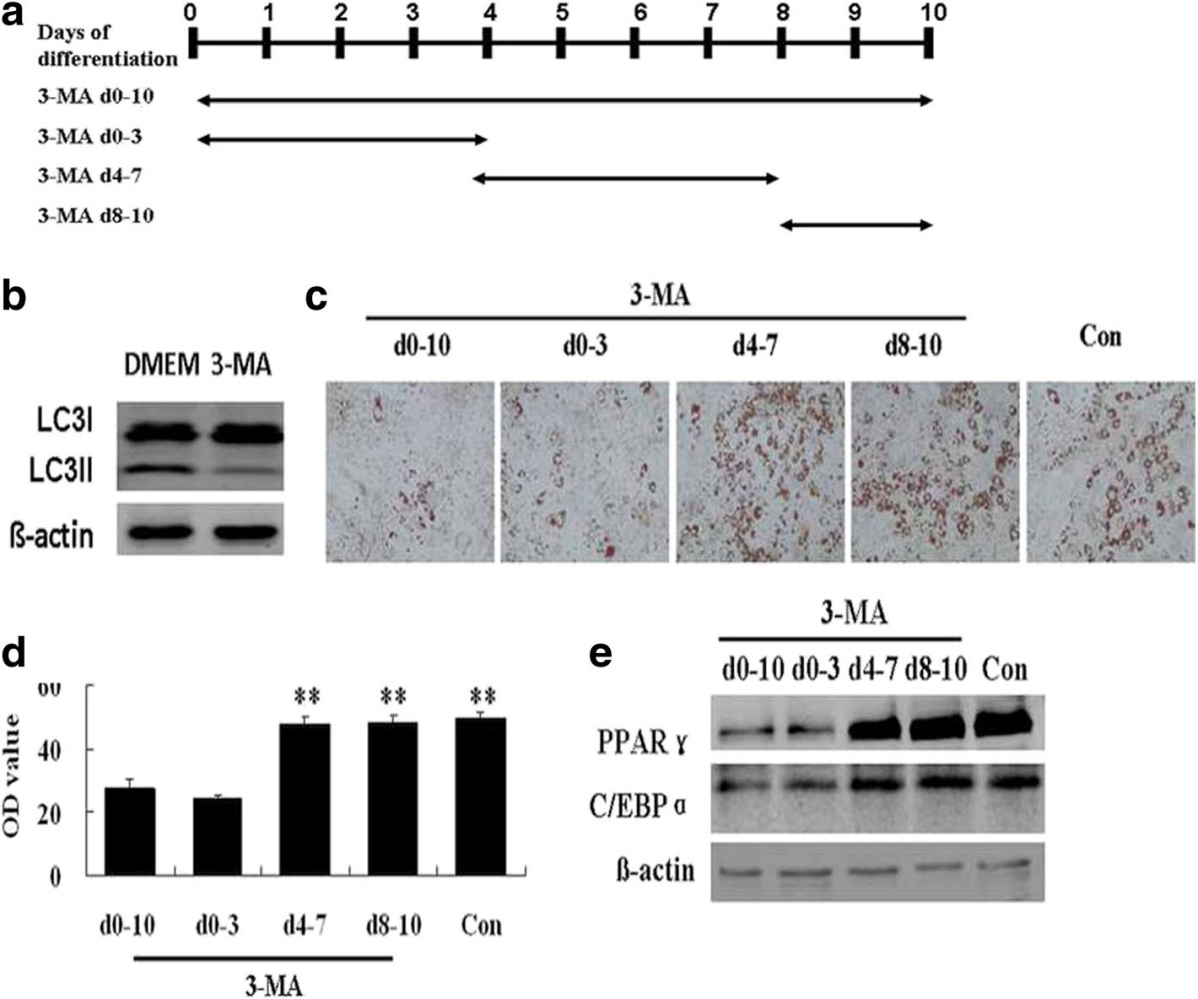
Effects of autophagy inhibition at different stages of differentiation on oleic acid (OA)-induced adipogenesis in hADSCs. **a** Adipogenic differentiation in hADSCs was induced with 80 μM OA, and the cells were treated with 3MA (10 mM) for different periods of time. **b** Western blot analysis of LC3II/I in hADSCs treated with 3MA for 24 h. **c** Oil red O staining at the end of differentiation (day 10). **d** Quantification of intracellular lipids at day 10. **e** Western blot analysis of PPARγ and C/EBPα at day 10. Data are shown as the mean ± SD (*n* = 3). ^**^p < 0.01 vs. control

We investigated the effects of autophagy inhibition on adipogenesis in hADSCs with 3-methyladenine (3MA) at different stages of differentiation (Fig. 3a). The conversion of LCI to LCII in hADSCs was significantly decreased when the cells were incubated with 10 mM 3MA for 24 h (Fig. 3b), indicating that treatment with 3MA could successfully inhibit autophagy. In addition, adipogenesis, in terms of oil red O staining and the intracellular lipid content, was significantly reduced by treatment with 3MA during the early stage (days 0–3) as well as throughout the whole differentiation process (days 0–10), compared with the control group (Fig. 3c–d). However, inhibition of autophagy with 3MA during the middle stage (days 4–7) or the late stage (days 8–10) of differentiation did not affect OA-induced adipogenesis in hADSCs (Fig. 3c–d). In addition, changes in the protein expression of PPARγ and C/EBPα showed a similar pattern as the oil red O staining and intracellular lipid analysis results (Fig. 3e). These findings suggest that autophagy may play a vital role at the early stage of adipogenic differentiation in hADSCs.

### Knockdown of p62 enhanced OA-induced adipogenesis of hADSCs

**Fig. 4:**
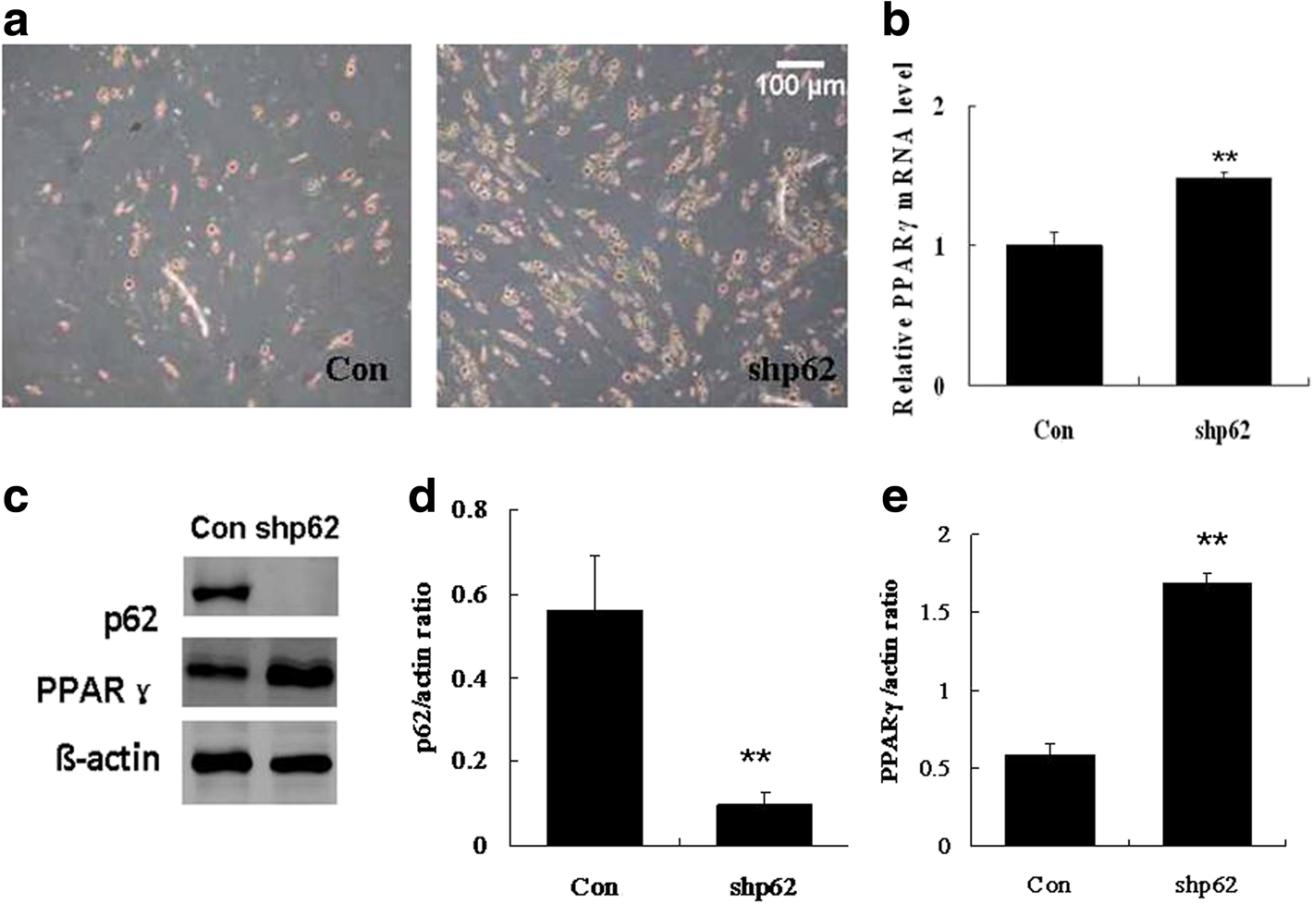
Knockdown of p62 enhanced adipogenesis of hADSCs. hADSCs were infected with shp62 for 72 h, and then adipogenic differentiation was induced with 80 μM oleic acid (OA). **a** Intracellular lipids were stained with oil red O at the end of differentiation (day 10). **b** Gene expression of PPARγ at day 10. **c** Western blot of p62 and PPARγ at day 10. **d** Quantification of p62 protein expression. **e** Quantification of PPARγ protein expression. Values are the mean ± SD (*n* = 3). **p < 0.01 vs. control

The phagophore elongates and engulfs autophagic cargo, whose recognition is mediated by adaptor proteins such as p62, and finally the phagophore closes to form an autophagosome [19, 20]. p62 is expected to play a key role in adipogenic differentiation of hADSCs. Therefore, in this study, we investigated the function of p62 in adipogenic differentiation of hADSCs by knocking it down with a lentiviral p62 shRNA (shp62). The results showed that the oil red O-positive cells (Fig. 4a) as well as the mRNA (Fig. 4b) and protein (Fig. 4c and e) levels of PPARγ at day 10 of differentiation were significantly increased after knockdown of p62 in hADSCs, indicating that knockdown of p62 promoted OA-induced adipogenesis in hADSCs. As expected, the protein expression of p62 was substantially decreased after treatment with shp62 (Fig. 4c–d).

### OA increased mitochondrial content during adipogenic differentiation

**Fig. 5:**
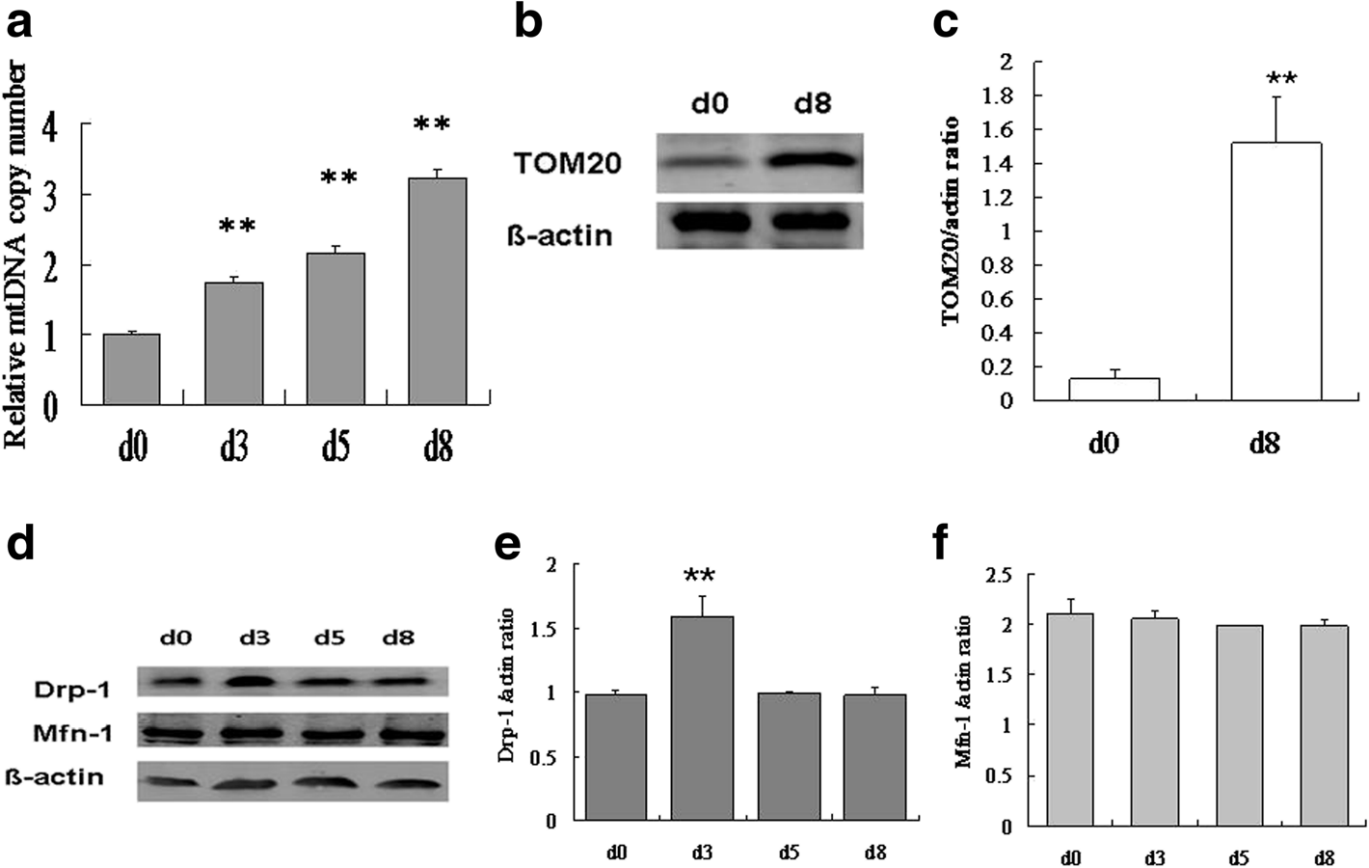
The number and fusion–fission balance of mitochondria in hADSCs during adipogenic differentiation. **a** The mtDNA (Nd1 gene) copy number was measured by quantitative PCR at days 0, 3, 5, and 8 of adipogenic differentiation. Western blot (**b**) and quantification (**c**) of the mitochondrial membrane protein TOM20. **d** Western blot of DRP-1 and MFN-1. **e** Quantification of the DRP-1 protein expression level. **f** Quantification of the MFN-1 protein expression level. Values are the mean ± SD (*n* = 3). **p < 0.01 vs. d0

It is not yet known whether mitophagy is induced during adipogenesis or whether a general increase in nonspecific autophagic degradation of mitochondria affects adipogenesis [21]. To investigate the mitochondrial content in OA-induced adipogenic differentiation, we measured the copy number of mtDNA (ND1 gene) by quantitative PCR and the protein level of TOM20, a mitochondrial membrane protein, by western blot analysis. As shown in Fig. 5a, the ND1 DNA copy number was increased following OA treatment. Compared with baseline (day 0), the protein expression of TOM20 was significantly increased in differentiated cells at day 8 (*p* < 0.01, Fig. 5b–c). These results showed that mitochondrial content was significantly increased at the end of differentiation and that the energy-producing ability was further improved by the increased mitochondrial membrane protein level. The quality of mitochondria was increased greatly by mitophagy, which ensured the refreshment of mitochondria. Therefore, we further investigated the correlative index of mitophagy during adipogenic differentiation. Mitochondrial fusion and fission have a close relationship with mitophagy, so we measured the protein expression of the mitochondrial fission protein DRP-1 and the fusion protein MFN-1 during adipogenesis. No notable change in expression of MFN-1 occurred during adipogenesis (Fig. 5d and f). Interestingly, DRP-1 was upregulated at day 3, but it returned to the baseline level on day 5 and day 8 (Fig. 5d–e). These data showed that the balance between mitochondrial fusion and fission was broken and that the mitochondria tended to become instable, indicating that mitophagy was increased during this period.

### Knockdown of p62 enhanced OA-induced adipogenesis of hADSCs by increasing mitophagy

**Fig. 6:**
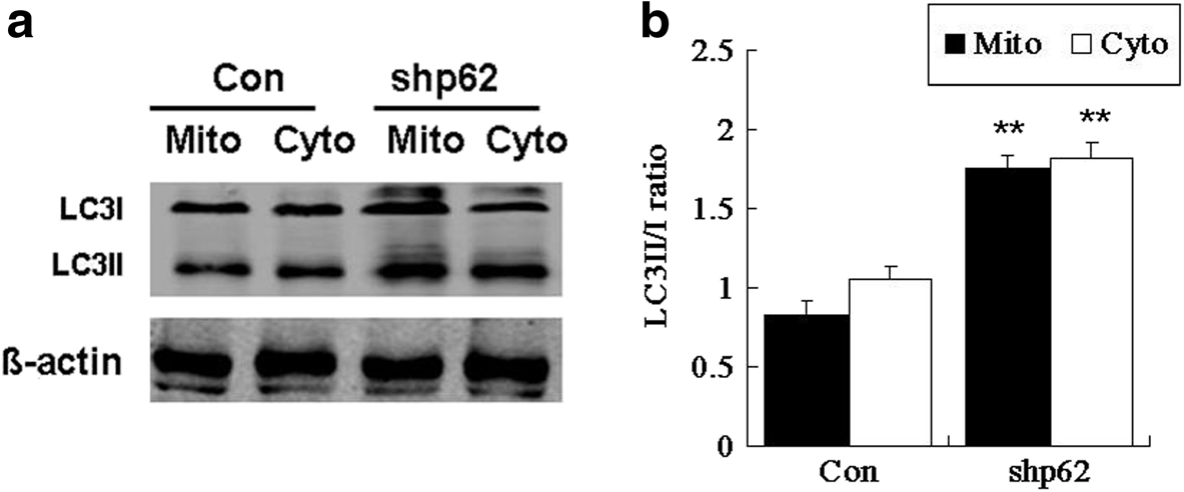
Knockdown of p62 promoted mitophagy at the early stage of adipogenesis in hADSCs. **a** Western blot of mitochondrial and cytoplasmic LC3II/I at day 3 of adipogenic differentiation in hADSCs treated with shp62 or control shRNA. **b** Quantification of the mitochondrial and cytoplasmic LC3II/I ratios. Values are the mean ± SD (*n* = 3). ^**^p < 0.01 vs. control

Since autophagy played a role mainly in the early stage of adipogenesis, the following parameters were detected at day 3 of differentiation. After mitochondrial autophagy occurred, mitochondria combined to form autophagosomes. Mito-LC3II/I was detected as a marker of mitophagy. Meanwhile, cyto-LC3II/I was also measured as a marker of autophagy. Western blot analysis showed that knockdown of p62 increased the mito-LC3II/I ratio by 110.1% and the cyto-LC3II/I ratio by 73.3% (Fig. 6a–b). These results indicated that knockdown of p62 promoted mitophagy during OA-induced adipogenesis in hADSCs. p62 knockdown-induced mitophagy is unlikely secondary to autophagy as there was a trend towards a higher mito-LC3II/I ratio than the cyto-LC3II/I ratio.

**Fig. 7:**
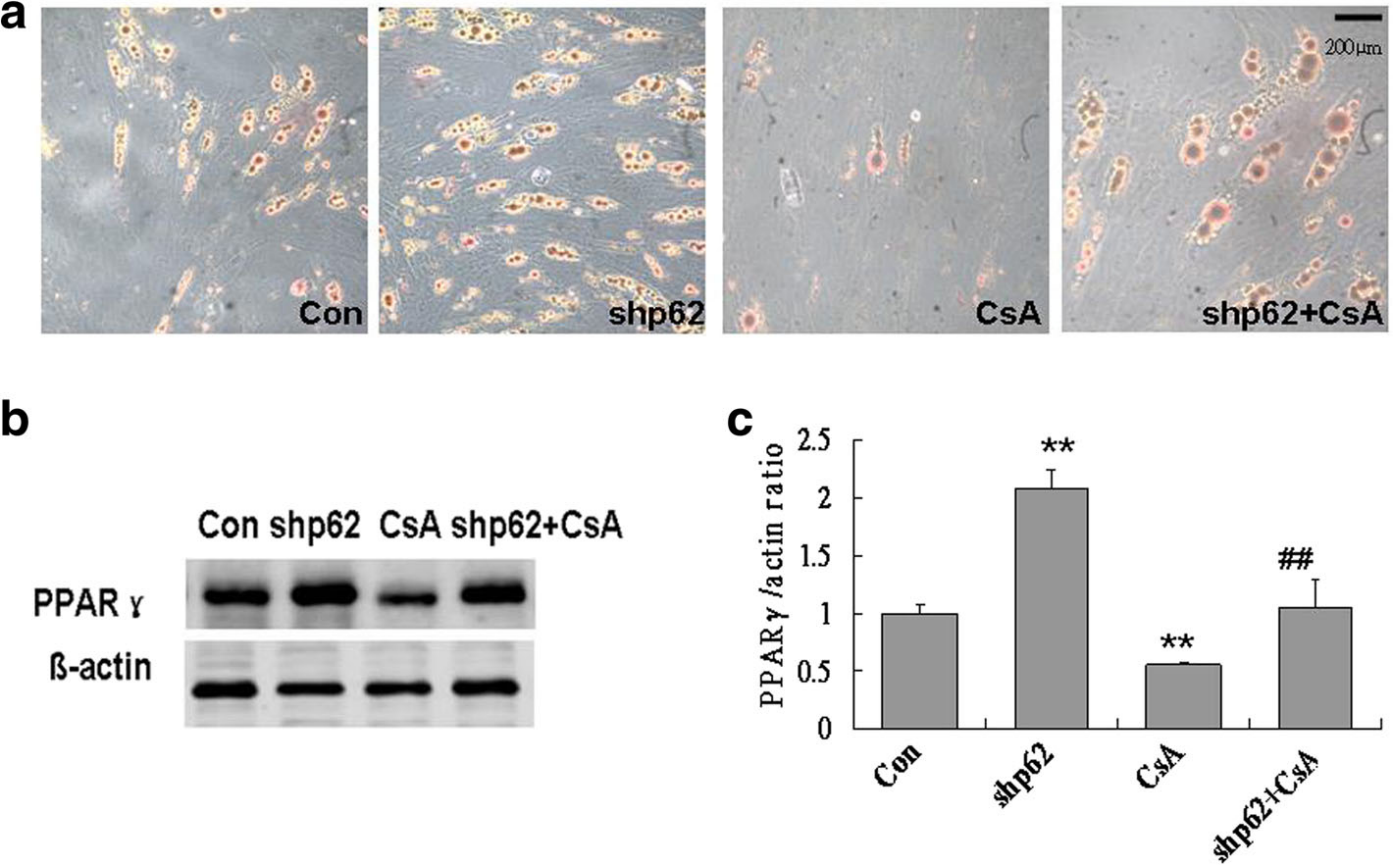
Inhibition of mitophagy abolished p62 knockdown-enhanced adipogenesis of hADSCs. hADSCs were pretreated with shp62 for 72 h and then induced by 80 μM oleic acid (OA) with or without 5 μM cyclosporine A (CsA), a mitophagy inhibitor. Differentiated cells were analyzed on day 10. **a** Oil red O staining. **b** Western blot of PPARγ. **c** Quantification of PPARγ protein expression. Values are the mean ± SD (*n* = 3). **p < 0.01 vs. control, ^##^*p* < 0.01 vs. shp62

To further investigate whether the increase of mitophagy caused by p62 silencing is related to the increase of adipogenesis, cyclosporine A, a selective inhibitor of mitochondrial autophagy, was used at the early stage of differentiation (days 0–3). The results showed that p62 knockdown-induced increases in oil red O-positive cells and protein expression of PPARγ were abolished by treatment with cyclosporine A (Fig. 7a–c). These data suggested that p62 knockdown-enhanced adipogenesis may be dependent on mitophagy at the early stage of differentiation and that p62 may affect adipogenesis of hADSCs through regulating mitophagy.

## Discussion

Obesity and insulin resistance have been observed in p62 gene-knockout mice, and the basal lipolytic hydrolysis is slower than that of wild-type mice. In addition, the number of intracellular lipid droplets, synthesis of triglycerides, and the size of adipocytes are increased in p62 gene-knockout mice [13]. In the present study, knockdown of p62 significantly enhanced OA-induced adipogenesis in hADSCs. These results were similar to those observed in p62 gene-knockout mice, suggesting that loss of p62 expression increases adipogenic differentiation and that p62 plays an important role in human adipocyte differentiation. Therefore, it is speculated that p62 may play a protective role in the development of obesity that is caused by an increased number of adipocytes.

OA-induced adipogenesis is probably due to increased FFA uptake. In this study, we found that OA increased the protein expression of the adipogenesis-related gene PPARγ, which promotes FFA uptake into adipocytes. Yu et al. have reported that OA and linoleic acid promote lipid accumulation in adipocytes, probably through regulating PPARγ phosphorylation [22], which is consistent with our results.

It has been reported that autophagy is involved in the differentiation of 3 T3-L1 pre-adipocytes [23, 24]. In the present study, we observed that OA-induced adipogenesis and autophagic activity changed at different stages of differentiation in hADSCs. The results showed that autophagy in hADSCs was at a very low level at baseline, which is conducive to the maintenance of homeostasis. After adipogenic induction with OA, the autophagy level was significantly increased at the early stage, and then it decreased at the later stage of adipogenic differentiation. Inhibition of autophagy using 3MA at the early stage resulted in a significant decrease in adipogenesis, while 3MA treatment at the middle or late stage did not affect adipogenesis. These findings suggest that autophagy is required for replacing cellular components during adipogenic differentiation. Otherwise, it would be difficult to achieve the switch of cellular components and functional transformation, which would enable the cells to maintain their original undifferentiated state. Interestingly, autophagy is crucial during the initiation of adipogenesis. A possible reason is that the relevant proteins that determine the new phenotype and function of the cells are mainly transformed at the early stage of adipogenesis. In contrast, the later stages of differentiation may be comprised of a cascade reaction, and the autophagic activity only needs to be maintained at a basal level. Recent studies have found that mitochondria play a vital role in self-renewal and directional differentiation of stem cells. When stem cells are differentiated, mitochondria gradually become “mature” and their number and function increase [25, 26]. The expression of the mitochondrial marker protein TOM20 and the mtDNA copy number can reflect mitochondrial content to a certain degree [27–29]. The results showed that both parameters were continuously increasing during OA-induced adipogenesis in hADSCs, which further verified the existing speculation. The increase of mitochondrial content during adipogenesis may have two functions. First, the process of cell differentiation requires mitochondria to provide enough ATP to enable the cells to undergo re-establishment. Second, mitochondria may be necessary for the synthesis of triglycerides and the formation of lipid droplets.

The results of this study showed that p62 knockdown enhanced mitophagy. The reason may be related to the duality of p62 on mitochondria: p62 does not only mediate the genesis of mitophagy, but it also stabilizes mitochondria from injury to prevent mitophagy. Based on our results, we speculated that knockdown of p62 in hADSCs resulted in a significant reduction of its stabilization function, resulting in increased mitophagy. Alternatively, knockdown of p62 increased the activity of other mediators to initiate mitophagy. In this case, p62 as a mediator for mitophagy is not necessary. In adipogenic differentiation of hADSCs, knockdown of p62 led to an uneven distribution of autophagic fluxes: a relatively increased mitophagy flux. Thus, the refreshment of a large number of mitochondria was ensured, which further promoted lipid synthesis and differentiation progression.

A limitation of this study was that heterogeneity of the primary cultured adipocytes may affect both adipogenesis and mitophagy. A previous study has demonstrated that the most commonly utilized adipose tissue extraction technique leads to a heterogeneous cell population [30]. In addition, primary cultured adipocytes partially retain the phenotypic properties of the tissue donor, such as gender, age, or body weight [14]. Moreover, the heterogeneity of mesenchymal stem cells in the final differentiation stage is variable. However, this variability is largely reduced under proinflammatory conditions or by knockdown of certain genes [31, 32]. In this study, knockdown of p62 in differentiating hADSCs reduced the heterogeneity of hADSCs. Nevertheless, further studies using homogeneous adipocytes are warranted.

## Conclusions

In combination with insulin and dexamethasone, OA can successfully induce adipogenesis of hADSCs. Autophagy plays a vital role at the early stage of adipogenesis; thus, knockdown of p62 may promote adipogenesis by increasing the activity of mitophagy.

## Abbreviations

3MA: 3-methyladenine
Atg5: Autophagy related 5
DRP-1: Dynamin-related protein 1
FFAs: Free fatty acids
hADSCs: Human adipose-derived stromal cells
IBMX: Isobutylmethylxantine
LC3: Microtubule-associated protein light chain 3
MFN-1: Mitofusin 1
ND1: NADH dehydrogenase subunit 1
OA: Oleic acid
PPARγ: Peroxisome proliferator activated receptor γ
TOM20: Mitochondrial outer membrane 20
